# Anatomical terminology of the internal nose and paranasal sinuses: cross-cultural adaptation to Portuguese^☆^

**DOI:** 10.1016/j.bjorl.2018.08.003

**Published:** 2018-09-18

**Authors:** Thiago Freire Pinto Bezerra, Aldo Stamm, Wilma Teresinha Anselmo-Lima, Marco Aurélio Fornazieri, Nelson D’Ávila Melo, Leonardo Balsalobre, Geraldo Pereira Jotz, Henrique Zaquia Leão, André Alencar Araripe Nunes, Alexandre Felippu, Antonio Carlos Cedin, Carlos D. Pinheiro-Neto, Diego Lima Oliveira, Eulalia Sakano, Eduardo Macoto Kosugi, Elizabeth Araújo, Fabiana Cardoso Pereira Valera, Fábio de Rezende Pinna, Fabrizio Ricci Romano, Francine Grecco de Melo Pádua, Henrique Faria Ramos, João Telles, Leonardo Conrado Barbosa de Sá, Leopoldo Marques D’Assunção Filho, Luiz Ubirajara Sennes, Luis Carlos Gregório, Marcelo H. Sampaio, Marco César Jorge dos Santos, Marco Franca, Marcos Mocellin, Marcus Miranda Lessa, Melissa Ameloti G. Avelino, Miguel Tepedino, Nilvano Alves de Andrade, Otavio B. Piltcher, Renato Roithmann, Renata Mendonça Pilan, Roberto Campos Meireles, Roberto Eustáquio Guimarães, Rodrigo de Paula Santos, Rogério Pezato, Shirley Pignatari, Tatiana Telles Abdo, Victor Nakajima, Washington Almeida, Richard L. Voegels

**Affiliations:** a Universidade Federal de Pernambuco (UFPE), Recife, PE, Brazil; b Universidade de São Paulo (USP), Faculdade de Medicina (FM), São Paulo, SP, Brazil; c Complexo Hospitalar Edmundo Vasconcelos, Centro de Otorrinolaringologia e Fonoaudiologia (COF), São Paulo, SP, Brazil; d Universidade de São Paulo (USP-RP), Faculdade de Medicina (FM), Ribeirão Preto, SP, Brazil; e Universidade Estadual de Londrina (UEL), Londrina, PR, Brazil; f Pontifícia Universidade Católica do Paraná (PUC-PR), Londrina, PR, Brazil; g Universidade Tiradentes (UNIT), Aracaju, SE, Brazil; h Universidade Federal do Rio Grande do Sul (UFRGS), Porto Alegre, RS, Brazil; i Universidade Federal do Ceará (UFC), Fortaleza, CE, Brazil; j Instituto Felippu de Otorrinolaringologia e Base do Crânio, São Paulo, SP, Brazil; k Beneficência Portuguesa de São Paulo, São Paulo, SP, Brazil; l Albany Medical Center, Albany, NY, United States; m Hospital Memorial Arthur Ramos (HMAR), Maceió, AL, Brazil; n Universidade Estadual de Campinas (UNICAMP), Campinas, SP, Brazil; o Universidade Federal de São Paulo (UNIFESP), Escola Paulista de Medicina (EPM), São Paulo, SP, Brazil; p Hospital Israelita Albert Einstein, São Paulo, SP, Brazil; q Universidade Federal do Espírito Santo (UFES), Vitória, ES, Brazil; r Universidade do Estado do Rio de Janeiro (UERJ), Faculdade de Ciências Médicas, Rio de Janeiro, RJ, Brazil; s Instituto Paranaense de Otorrinolaringologia, Curitiba, PR, Brazil; t Faculdade de Enfermagem e Medicina Nova Esperança (FAMENE), João Pessoa, PB, Brazil; u Universidade Federal do Paraná (UFPR), Curitiba, PR, Brazil; v Universidade Federal da Bahia (UFBA), Faculdade de Medicina, Salvador, BA, Brazil; w Universidade Federal de Goiás (UFG), Goiânia, GO, Brazil; x Policlínica Botafogo, Rio de Janeiro, RJ, Brazil; y Fundação Bahiana para Desenvolvimento das Ciências, Salvador, BA, Brazil; z Universidade Luterana do Brasil, Faculdade de Medicina, Porto Alegre, RS, Brazil; aa Universidade Federal do Rio de Janeiro (UFRJ), Rio de Janeiro, RJ, Brazil; bb Universidade Federal de Minas Gerais (UFMG), Belo Horizonte, MG, Brazil; cc Universidade Estadual Paulista (UNESP), Botucatu, SP, Brazil; dd Hospital Otorrinos de Feira de Santana, Feira de Santana, BA, Brazil

**Keywords:** Cross-cultural adaptation, Anatomy, Nose, Paranasal sinus, Consensus, Adaptação transcultural, Anatomia, Nariz, Cavidades paranasais, Consenso

## Abstract

**Introduction:**

Functional endonasal endoscopic surgery is a frequent surgical procedure among otorhinolaryngologists. In 2014, the European Society of Rhinology published the “European Position Paper on the Anatomical Terminology of the Internal Nose and Paranasal Sinuses”, aiming to unify the terms in the English language. We do not yet have a unified terminology in the Portuguese language.

**Objective:**

Transcultural adaptation of the anatomical terms of the nose and paranasal cavities of the “European Anatomical Terminology of the Internal Nose and Paranasal Sinuses” to Portuguese.

**Methods:**

A group of rhinologists from diverse parts of Brazil, all experienced in endoscopic endonasal surgery, was invited to participate in the creation of this position paper on the anatomical terms of the nose and paranasal sinuses in the Portuguese language according to the methodology adapted from that previously described by Rudmik and Smith.

**Results:**

The results of this document were generated based on the agreement of the majority of the participants according to the most popular suggestions among the rhinologists. A cross-cultural adaptation of the sinonasal anatomical terminology was consolidated. We suggest the terms “inferior turbinate”, “nasal septum”, “(bone/cartilaginous) part of the nasal septum”, “(middle/inferior) nasal meatus”, “frontal sinus drainage pathway”, “frontal recess” and “uncinate process” be standardized.

**Conclusion:**

We have consolidated a Portuguese version of the European Anatomical Terminology of the Internal Nose and Paranasal Sinuses, which will help in the publication of technical announcements, scientific publications and the teaching of the internal anatomical terms of the nose and paranasal sinuses in Brazil.

## Resumo

**Introdução:**

A cirurgia endoscópica funcional endonasal é um procedimento cirúrgico frequente entre os otorrinolaringologistas. Em 2014, a Sociedade Europeia de Rinologia publicou o “Documento Europeu para Posicionamento sobre a Terminologia Anatômica Interna do Nariz e das Cavidades Paranasais” com o objetivo de unificar os termos na língua inglesa. Ainda não dispomos de uma terminologia unificada na língua portuguesa.

**Objetivo:**

Adaptação transcultural dos termos anatômicos do nariz e das cavidades paranasais para o português da “*European Anatomical Terminology of the Internal Nose and Paranasal Sinuses*”.

**Método:**

Um grupo de rinologistas de todo o Brasil, com experiência em cirurgia endoscópica endonasal, foi convidado a participar da elaboração desse posicionamento sobre os termos anatômicos do nariz e das cavidades paranasais para o português conforme metodologia adaptada da previamente descrita por Rudmik e Smith.

**Resultados:**

Os resultados desse documento foram gerados a partir da concordância da maioria dos participantes conforme as sugestões mais populares entre os rinologistas. Uma adaptação transcultural da terminologia anatômica nasossinusal foi consolidada. Sugerimos que se busque uniformizar termos como “concha inferior”, “septo nasal”, “porção (óssea/cartilagionasa) do septo nasal”, “meato (médio/ inferior) nasal”, “via da drenagem do seio frontal”,“recesso frontal” e “processo uncinado”.

**Conclusão:**

Consolidamos uma versão adaptada em português da “European Anatomical Terminology of the Internal Nose and Paranasal Sinuses” que auxiliará a publicação de comunicados técnicos, publicações científicas e o ensino dos termos anatômicos internos do nariz e das cavidades paranasais no Brasil.

## Introduction

Endoscopic surgery and sinonasal computed tomography stimulated rhinology in the early 1980s into the revival of research in the fields of anatomy and physiology of the nose and paranasal sinuses.^1^ In 1994, the International Conference on Sinus Disease took place aiming to describe the newly identified structures in detail, since Anatomical Terminology had few descriptors of the sinonasal anatomy.^2^, ^3^

In 2014, the European Society of Rhinology published the “European Position Paper on the Anatomical Terminology of the Internal Nose and Paranasal Sinuses” to unify the sinonasal anatomical terminology through the review of anatomical terms and analysis of the official “Anatomical Terminology”.^1^ They sought to respect the embryological development of structures, avoid terminology in Latin, remove eponyms, and simplify the anatomical terms.

Lund et al. summarized in the English language all structures that could be found during a routine sinonasal endoscopic surgery. At that time, there were several publications on clinical anatomy and much discussion about the exact names and definitions for structures of surgical relevance.^2^

It is clearly necessary to unify this terminology in all other languages and, that in the process of cross-cultural adaptation, the defined terms find correspondence in English. This publication in other languages would facilitate technical information, scientific publications and the teaching of the internal anatomical terms of the nose and paranasal cavities.

The purpose of this study is the cross-cultural adaptation of the anatomical terms of the nose and paranasal cavities to the Portuguese language of the European Anatomical Terminology of the Internal Nose and Paranasal Sinuses and the proposition of a Sinonasal Anatomical Terminology in Portuguese.

## Methods

**Figure 1:**
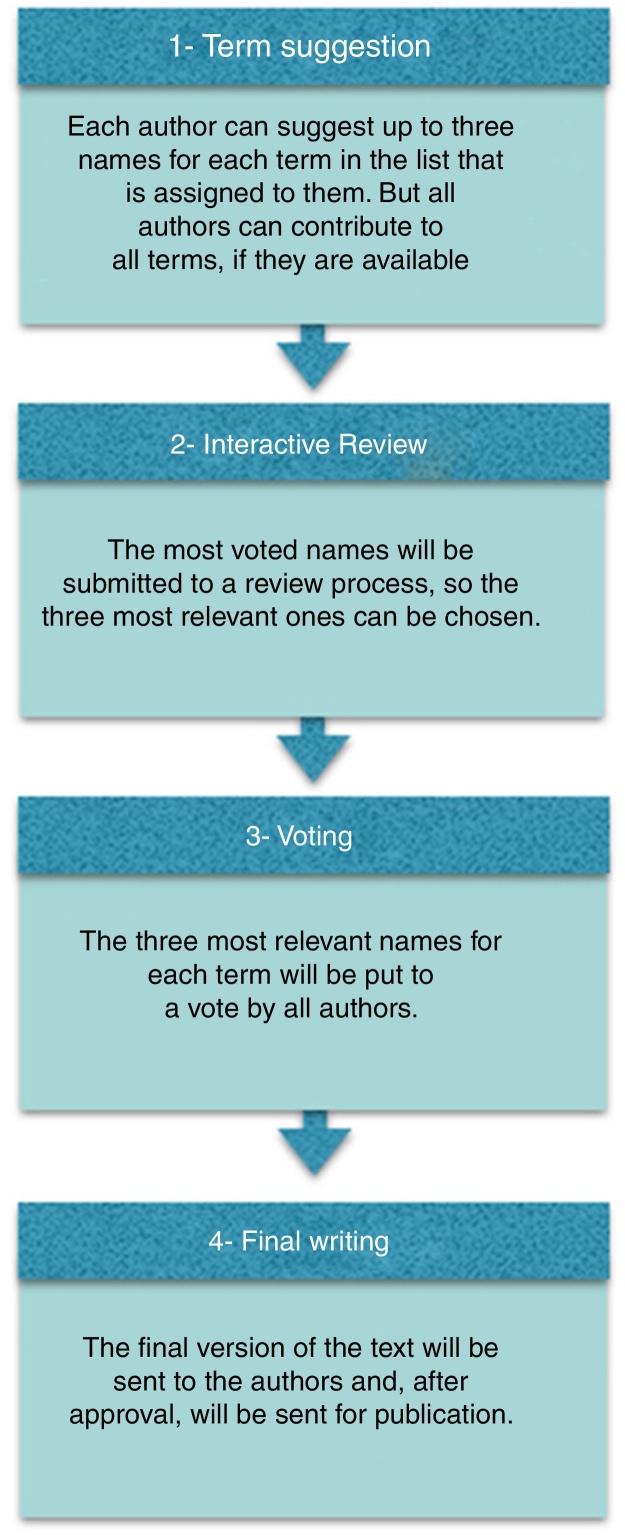
Study design.

This is a prospective study of cross-cultural adaptation, carried out in Brazil, from 2015 to 2016. Forty-four acknowledged rhinologists from all over Brazil were invited to participate (Fig. 1). We followed an adapted version of the method used by Rudmik and Smith.^4^ The entire study process was carried out at distance with the aid of a platform, which allowed the unification and analysis of the results.

### Inclusion criteria for the group of rhinologists

- Voluntary interest in participating in the study;
- Otorhinolaryngologists with proven experience in sinonasal endoscopic surgery through publications on the subject and/or working in referral centers in rhinology.

### Exclusion criteria for the group of rhinologists

- No interest in participating after the invitation;
- No experience in sinonasal endoscopic surgery through publications on the subject and/or not working in referral centers in rhinology.

### Phase 1. Term suggestion phase

The final list of the 126 terms of Supplement 24 (1) was divided into 8 blocks (Table 1) and each block was randomly assigned to a group of 4–5 authors. All authors received a copy of the original supplement by e-mail^1^ and were invited to suggest three or more known terms in Portuguese for each English term of the block assigned to them. All authors were also encouraged to suggest more terms for other blocks.

**Table 1:** Final results of the suggested terms and voting frequency of the three voted terms.

Suggested term (EPOS)	Terminologia Anatômica	3 Termos mais sugeridos por ordem de frequências e termo proposto (sublinhado)		
“Nasal cavity”	*Cavitas nasi*	Cavidade nasal 82,9% (34/41)^a^	Fossa nasal 14,6% (6/41)	Cavidade do nariz 2,4% (1/41)
“Lateral nasal wall”	*Não existe (n.e.)*	Parede nasal lateral 41,5% (17/41)^a^	Parede lateral da cavidade nasal 39%(16/41)	Parede lateral do nariz 19,5% (8/41)
“Nasal floor”	*n.e.*	Assoalho da Cavidade nasal 48,8% (20/41)^a^	Assoalho nasal 29,3% (12/41)	Assoalho da fossa nasal 22% (9/41)
”Nasal septum”	*Septum nasi*	Septo nasal 97,6% (40/41)^a^	Septo do nariz 2,4%(1/41)	
“Septal cartilage”	*Pars cartilaginea (septi nasi) Cartilago septi nasi*	Cartilagem septal 61% (25/41)^a^	Septo nasal cartilaginoso 24,4% (10/41)	Cartilagem nasal septal 14,6% (6/41)
“Bony septum”	*Pars ossea septi nasi*	Septo nasal ósseo 48,8% (20/41)	Porção óssea do Septo nasal 34,1% (14/41)^a^	Septo ósseo 17,1 (7/41)%
“Perpendicular plate of ethmoid”	*Lamina perpendicularis ossis ethmoidalis*	Lamina perpendicular do etmóide 73,2% (30/41)^a^	Lâmina perpendicular do osso etmóide 24,4% (10/41)	Lâmina Óssea Septal 2,4% (1/41)
“Vomer”	*Pars ossea septi nasi; Vomer*	Vômer 87,8% (36/41)^a^	Vômer nasal 12,2% (5/41)	Osso Inferior do Septo Nasal 0,0%
“Membranous portion (of nasal septum)”	*Pars membranacea septi nasi*	Porção membranosa do septo nasal 53,7% (22/41)^a^	Septo nasal membranoso 36,6% (15/41)	Septo membranoso 9,8% (4/41)
“Vomero-nasal organ”	*Organum vomeronasale*	Órgão vômero-nasal 51,2% (21/41)^a^	Órgão vomeronasal 48,8% (20/41)	
“Septal tubercle”	*não existe*	Tuberculo septal 70,7% (29/41)^a^	Tuberculo nasal 19,5% (8/41)	Corpo cavernoso do septo nasal 9,8% (4/41)
“Inferior turbinate”	*Concha nasi inferior*	Concha nasal Inferior 61% (25/41)	Concha inferior 39% (16/41)^a^	Turbina nasal inferior 0,0%
“Inferior meatus”	*Meatus nasi inferior*	Meato inferior 53,7% (22/41)	Meato nasal inferior 43,9% (18/41)^a^	Meato inferior nasal 2,4% (1/41)
“Naso-lacrimal duct opening”	*Apertura/ostium ductus nasolacrimalis*	Abertura do Ducto nasolacrimal 51,2% (21/41)^a^	Óstio do ducto nasolacrimal 48,8% (20/41)	Abertura nasolacrimal 0,0% (0/41)
“Middle turbinate”	*Concha nasi media*	Concha média 51,2% (21/41)^a^	Concha nasal média 48,8% (20/41)	Turbina nasal média 0,0% (0/41)
“Basal lamella of middle turbinate”	*n.e*	Lamela basal da concha media 53,7% (22/41)^a^	Lamela basal da concha media 31,7% (13/41)	Lamela basal 14, 6% (6/41)
“Paradoxical middle turbinate”	*n.e.*	Concha média paradoxal 61% (25/41)^a^	Concha nasal média paradoxal 36,6% (15/41)	Concha nasal média com curvatura paradoxal 2,4% (1/41)
“Concha bullosa (of middle turbinate)”	*n.e.*	Concha média bolhosa 82,9% (34/41)^a^	Concha bolhosa 17,1% (7/41)	Concha média globose 0,0% (0/41)
“Interlamellar cell”	*n.e.*	Célula interlamellar 78% (32/41)^a^	t.b.a 22% (9/41)	
“Middle meatus”	*Meatus nasi medius*	Meato médio 48,8% (20/41)	Meato médio nasal 34,1% (14/41)	Meato nasal médio 17,1% (7/41)^a^
“Ostiomeatal complex”	*n.e.*	Complexo óstio-meatal 85,4% (35/41)^a^	Unidade óstiomeatal 14,6% (6/41)	
“Superior turbinate”	*Concha nasi superior*	Concha nasal superior 51,2% (21/41)	Concha superior 48,8% (20/41)^a^	
“Concha bullosa (of superior turbinate)”	*n.e.*	Concha superior bolhosa 53,7% (22/41)^a^	Concha nasal superior bolhosa 39% (16/41)	Concha superior pneumatizada 7,3%(3/41)
“Superior meatus”	*Meatus nasi superior*	Meato superior 48,8% (20/41)	Meato nasal superior 41,5% (17/41)^a^	Meato superior nasal 9,8% (4/41)
“Supreme turbinate”	*Concha nasi suprema*	Concha nasal suprema 51,2% (21/41)	Concha suprema 48,8% (20/41)^a^	
“Paradoxical middle turbinate”	*n.e.*	Meato supremo nasal 48,8% (20/41)	Meato supremo 48,8% (20/41)	Meato nasal supremo 2,4% (1/41)^a^
“Spheno-ethmoidal recess”	*Recessus sphenoethmoidalis*	Recesso esfenoetmoidal 73,2% (30/41)^a^	Recesso esfeno-etmoidal 26,8% (11/41)	
“Sphenopalatine foramen”	*Foramen sphenopalatinum*	Forame esfenopalatino 82,9% (34/41)^a^	Forame da Artéria esfenopalatina 17,1% (7/41)	
“Olfactory cleft”	*Sulcus olfactorius*	Fenda olfatória 70,7% (29/41)^a^	Sulco olfatório 17,1% (7/41)	Área olfatória ou olfativa 12,2% (5/41)
“Olfactory rbre(s)”	*Fila olfactoria (Sing.: rlum olfactorium)*	Fibra(s) olfatória(s) 48,8% (20/41)	Fibras do nervo olfatório 46,3%(19/41)^a^	Nervos olfatórios 4,9% (2/41)
“Choana”	*Choana (Plur.: choanae); Apertura nasalis posterior*	Coana (coanas) 97,6% (20/41)^a^	Abertura nasal posterior 2,4% (1/41)	
“Maxillary sinus”	*Sinus maxillaris*	Seio maxilar 95,1% (19/41)^a^	Cavidade Paranasal Maxilar 4,9% (2/41)	
“Maxillary sinus ostium”	*n.e.*	Óstio do Seio maxilar 70,7% (29/41)^a^	Óstio natural do seio maxilar 29,3% (12/41)	Óstio da Cavidade Paranasal Maxilar 0,0%
“Accessory ostium”	*n.e*	Óstio acessório do seio maxilar 87,8% (36/41)^a^, ^b^	Óstio supranumerário do seio maxilar 7,3% (3/41)	Óstio acessório da Cavidade Paranasal Maxilar 4,9% (2/41)
“Maxillary hiatus”	*Hiatus maxillaris*	Hiato maxilar 97,6% (40/41)^a^	Tba 2,4% (1/41)	
“Infraorbital canal”	*Canalis infraorbitalis*	Canal do nervo infraorbitário 61% (25/41)^a^	Canal infraorbitário 39% (16/41)	Canal do nervo infra orbital 0,0% (0/41)
“Zygomatic recess”	*n.e.*	Recesso zigomático 87,8% (36/41)^a^	Recesso do osso zigomático 12,2% (5/41)	
“Alveolar recess”	*n.e.*	Recesso alveolar 68,3% (28/41)^a^	Processo alveolar 31,7% (13/41)	
“Prelacrimal recess”	*n.e.*	Recesso pré-lacrimal 100% (41/41)^a^		
“Lacrimal eminence”	*n.e.*	Eminência lacrimal 58,5% (24/41)^a^	Proeminência do osso lacrimal 41,5% (17/41)	
“Canine fossa”	*Fossa canina*	Fossa canina 100% (41/41)^a^		
“Anterior fontanelle”	*n.e.*	Fontanela anterior 100% (41/41)^a^		
“Posterior fontanelle”	*n.e*	Fontanela posterior 100% (41/41)^a^		
“Maxillary artery”	*Arteria maxillaris*	Artéria maxilar 78% (32/41)^a^	Artéria maxilar interna 22% (9/41)	
“Ethmoidal complex”	*Cellulae ethmoidales*	Células etmoidais 70,7% (29/41)	Complexo etmoidal 26,8% (11/41)^a^	Labirinto etmoidal 2,5% (1/41)
“t.b.a.”	*Cellulae ethmoidales mediae*	Células etmoidais medias 53,7% (22/41)	t.b.a. - a ser abandonado 43,9% (18/41)^a^	Lamela basal da Concha superior 2,4% (1/41)
“Posterior ethmoidal cells”	*Cellulae ethmoidales posteriores*	Células etmoidais posteriors 95,1% (39/41), substituído por Complexo Etmoidal Posterior^b^, ^a^	Etmóide posterior 4,9% (2/41)	Células do etmóide posterior 0,0% (0/41)
“Anterior ethmoidal artery”	*Arteria ethmoidalis anterior*	Artéria etmoidal anterior 100% (41/41)^a^		
“Accessory ethmoidal artery”	*n.e.*	Artéria etmoidal acessória 58,5% (24/41)^a^	Artéria etmoidal média 39%(16/41)	Artéria etmoidal intermedia 2,5% (1/41)
“Posterior ethmoidal artery”	*Arteria ethmoidalis posterior*	Artéria etmoidal posterior 100% (41/41)^a^		
“Anterior ethmoidal complex”	*Cellulae ethmoidales anteriores*	Células etmoidais anteriores 82,9% (34/41)	Complexo etmoidal anterior 12,2% (5/41)^a^	Seio etmoidal anterior 4,9% (2/41)
“Agger nasi”	*Agger nasi*	Agger Nasi 100% (41/41)^a^		
“Agger nasi cell”	*n.e. (cellula ethmoidalis anterior)*	Célula Agger Nasi 92,7% (38/41)^a^	Célula do Agger nasi 2,43% (1/41)	Agger nasi 2,43% (1/41)
“Uncinate process”	*Processus uncinatus*	Processo uncinado 78% (32/41)^a^	Processo unciforme 19,5% (8/41)	Uncinado 2,5% (1/41)
“Everted uncinate process”	*n.e.*	Processo uncinado evertido 78%(32/41)^a^	Processo unciforme evertido 22%(9/41)	
“Aerated uncinate process”	*n.e.*	Processo uncinado pneumatizado 80,5% (33/41)^a^	Processo unciforme pneumatizado 19,5%(8/41)	
“Basal lamella of uncinate process”	*n.e.*	Lamela basal do processo uncinado 80,5% (33/41)^a^	Lamela basal do processo unciforme 19,5% (8/41)	
“Inferior semilunar hiatus”	*Hiatus semilunaris*	Hiato semilunar inferior 90,2% (37/41)^a^	Hiato semilunar anterior 9,8% (4/41)	
“Superior semilunar hiatus”	*n.e.*	Hiato semilunar superior 85,4% (35/41)^a^	Recesso retrobular 14,6% (6/41)	
“Ethmoidal bulla”	*Bulla ethmoidalis*	Bula etmoidal 92,7% (38/41)^a^	Bolha etmoidal 7,3% (3/41)	
“Basal lamella of ethmoidal bulla”	*n.e.*	Lamela basal da Bula etmoidal 87,8% (36/41)^a^	Lamela basal da Bolha etmóidal 7,3% (3/41)	TA 2,4% (1/41)
“Suprabullar recess”	*n.e.*	Recesso suprabular 95,1% (39/41)^a^	Recesso supra-bolhoso 4,9% (2/41)	
“Retrobullar recess”	*n.e.*	Recesso retrobular 95,1% (39/41)^a^	Recesso retro-bolhoso 4,9% (2/41)	
“Supraorbital recess”	*n.e.*	Recesso supra-orbitário 95,1% (39/41)^a^	Recesso supra-orbital 2,45% (1/41)	Incisura supra-orbitaria 2,45% (1/41)
“Infraorbital cell”	*n.e.*	Célula infra-orbital 90,2% (37/41)^a^	Célula infra orbitaria 2,4% (1/41)	célula infraorbitária 2,4% (1/41)
“Ethmoidal infundibulum”	*Infundibulum ethmoidale*	Infundíbulo etmoidal 78% (32/41)^a^	Infundíbulo 22% (9/41)	
“Terminal recess”	*n.e*	Recesso terminal 100% (41/41)^a^		
“Frontal recess”	*n.e*	Recesso frontal 97,6% (40/41)^a^	Recesso do Seio frontal 2,4% (1/41)	
“t.b.a.”	*Ductus nasofrontalis*	Ducto nasofrontal 68,3% (28/41)	t.b.a. 29,3% (12/41)	Crista maxilar 2,4% (1/41)
“Lacrimal bulge”	*n.e.*	A ser abandonado 41,5% (17/41)	Crista maxilar 39% (16/41)	Ducto nasofrontal 19,5% (8/41)
“Ethmoidal crest”	*Crista ethmoidalis*	Crista etmoidal 73,2% (30/41)	Crista etmoidal do osso palatino 26,8% (11/41)	
“Frontal sinus drainage pathway”	*n.e*	Recesso frontal 63,4% (26/41)	Via da drenagem do Seio frontal 26,8% (11/41)^a^	Drenagem do Seio frontal 9,8% (4/41)
“Frontal sinus”	*Sinus frontalis*	Seio frontal 95,1% (39/41)	Cavidade paranasal Frontal 2,45% (1/41)	Cavidade frontal 2,45% (1/41)
“Frontal intersinus septum”	*Septum sinuum frontalium*	Septo intersinusal do seio frontal 92,7% (38/41)	Septo interfrontal 7,3% (3/41)	
“Frontal sinus infundibulum”	*n.e.*	Infundíbulo do seio frontal 63,4% (26/41)	a ser abandonado 29,3% (12/41)	Infundíbulo frontal 7,3% (3/41)
“Frontoethmoidal cells”	*Bullae frontales (sing.: bulla frontalis)*	Células frontoetmoidais 97,6% (40/41)	Células intrafrontais 2,4% (1/41)	
“Intersinus septal cell”	*n.e.*	Celula septal intersinusal 63,4% (26/41)	Célula interfrontal 34,1% (14/41)	Célula do septo sinusal 2,5% (1/41)
“a ser abandonado”	*n.e. (cellula ethmoidalis anterior)*	a ser abandonado 85,4% (35/41)	Bula frontal 14,6% (6/41)	
“Frontal sinus opening”	*Apertura sinus frontalis*	Óstio do Seio Frontal 48,8% (20/41)	Abertura do Seio frontal 34,1% (14/41)	Recesso frontal 17,1% (7/41)
“Frontal beak”	*Spina frontalis (ossis frontalis*	Espinha Frontal 41,5% (17/41)	Bico frontal 34,1% (14/41)	Espinha nasal superior 24,4% (10/41)
“Posterior ethmoidal complex”	*Cellulae ethmoidales posteriores*	Células etmoidais posteriors 80,5% (33/41)	Seio etmoidal posterior 9,8% (4/41)	Complexo Etmoidal Posterior 9,8% (4/41)
“Sphenoethmoidal cell”	*n.e. (cellula ethmoidalis posterior)*	Célula esfeno-etmoidal 48,8% (20/41)	Célula de Onodi 43,9% (18/41)	Célula etmoidal posterior 7,3% (3/41)
“Basal lamella of superior turbinate”	*n.e*	Lamela basal da concha nasal superior 100% (41/41)		
“Lamina papyracea”	*Lamina orbitalis ossis ethmoidalis*	Lâmina papirácea da parede medial da órbita 87,8% (36/41)	Parede medial da órbita 12,2% (5/41)	
“Orbital apex”	*n.e*	Ápice orbitário 100% (41/41)		
“Annulus of Zinn”	*Annulus tendineus communis*	Anel tendinoso comum 58,5% (24/41)	Ânulo orbital 41,5% (17/41)	
“Ophthalmic artery”	*Arteria ophthalmica*	Artéria Oftálmica 100% (41/41)		
“Sphenoid sinus”	*Sinus sphenoidalis*	Seio Esfenoidal 97,6% (40/41)	Cavidade Esfenoidal 2,4% (1/41)	
“Sphenoid intersinus septum”	*Septum sinuum sphenoidalium*	Septo Interesfenoidal 56,1% (23/41)	Septo intersinusal do seio esfenóidal 43,9% (18/41)	
“Sphenoid septations”	*n.e.*	Septos Intraesfenoidais 75,6% (31/41)	Septo intersinusal do seio esfenóidal 24,4% (10/41)	
“Sphenoid sinus ostium”	*Ostium (apertura) sinus sphenoidalis*	Óstio do seio esfenoidal 100%(41/41)	Óstio da cavidade esfenoidal 0,0%	
“Planum sphenoidale”	*Jugum sphenoidale*	Plano esfenoidal 100%(41/41)		
“Sellar loor”	*n.e.*	Assoalho da sela túrcia 73,2%(30/41)	Assoalho selar 26,8% (11/41)	
“Pterygoid (Vidian) canal”	*Canalis pterygoideus*	Canal pterigóideo 43,9% (18/41)	Canal do nervo vidiano 41,5% (17/41)	Canal do vidiano 14,6% (6/41)
“Foramen rotundum”	*Foramen rotundum*	Forame redondo 100% (41/41)		
“Lateral recess of sphenoid sinus”	*n.e.*	Recesso lateral do seio esfenoidal 100% (41/41)		
“Optic nerve tubercle”	*Tuberculum nervi optici*	Tubérculo do nervo óptico 97,6%(40/41)	Proeminência óssea do nervo óptico 2,4% (1/41)	
“Optic nerve canal”	*Canalis opticus*	Canal do nervo óptico 100%(41/41)		
“Carotid artery bulge”	*n.e. Proeminência da artérica carótida*	Proeminência da artéria carótida 92,7% (38/41)	Proeminência óssea da artéria carótida interna 2,4% (1/41)	Proeminência da artéria carótida 2,4% (1/41)
“Optico-carotid recess”	*n.e.*	Recesso óptico-carotídeo 100%(41/41)		
“Lateral craniopharyngeal (Sternberg ´s) canal”	*n.e.*	Canal lateral crânio-faríngeo 56,1% (23/41)	Canal de Sternberg 41,5% (17/41)	Canal crânio-faríngeo lateral 2,4% (1/41)
“Sphenoid rostrum”	*Rostrum sphenoidale*	Rostro do seio esfenoidal 100%(41/41)	Rostro da cavidade esfenoidal 0,0%	
“Vomerovaginal canal”	*Canalis vomerovaginalis*	Canal vomero-vaginal 97,6% (40/41)	Canal vomerovaginal 2,4% (1/41)	
“Palatovaginal canal”	*Canalis palatovaginalis*	Canal palato-vaginal 95,1% (39/41)	canal palatoesfenoidal 2,45% (1/41)	Canal palatovaginal 2,45% (1/41)
“Anterior cranial fossa”	*Fossa cranii anterior*	Fossa craniana anterior 75,6% (31/41)	Fossa anterior do crânio 24,4% (10/41)	Superfície anterior da base do crânio 0,0%
“Olfactory fossa”	*n.e*	Fossa olfatória 70,7% (29/41)	Área Olfatória 19,5% (8/41)	Goteira olfatória 9,8% (4/41)
“Cribriform plate”	*Lamina cribrosa (ossis ethmoidalis)*	Lâmina cribriforme 53,7% (22/41)	Placa cribriforme 29,3% (12/41)	Lâmina crivosa 17,1% (7/41)
“Cribriform foramina”	*Foramina cribrosa*	Forames cribriformes 58,5% (24/41)	Forames crivosos 22%(9/41)	Lâmina cribriforme 9,8% (4/41)
“Lateral lamella of cribriform plate”	*n.e.*	Lamela lateral da lâmina cribriforme 58,5% (24/41)	Lamela lateral da Placa Cribiforme 24,4% (10/41)	Lamela lateral da lâmina crivosa 17,1%(7/41)
“Ethmoidal roof”	*n.e.*	Teto do etmóide 61% (25/41)	Fóvea etmoidal 34,1% (14/41)	Fóvea etmoidal do osso frontal 4,9% (2/41)
“Crista galli”	*Crista galli*	Crista galli 95,1% (39/41)	Processo etmoidal 4,9% (2/41)	
“Pneumatized crista galli”	*n.e.*	Crista galli pneumatizada 95,1% (39/41)	Processo etmoidal pneumatizada 4,9% (2/41)	
“Foramen caecum”	*Foramen caecum*	Foramen cego 75,6% (31/41)	Foramen cecum 24,4% (10/41)	
“Middle cranial fossa”	*Fossa cranii media*	Fossa craniana media 80,5% (33/41)	Fossa media do crânio 14,6% (6/41)	Fossa media 4,9% (2/41)
“Sella (turcica)”	*Sella turcica*	Sela túrcica 92,7% (38/41)	Sela turca 4,9% (2/41)	Fossa pituitária 2,4% (1/41)
“Tuberculum sellae”	*Tuberculum sellae*	Tubérculo selar 65,9% (27/41)	Tubérculo da sela 34,1% (14/41)	
“Dorsum sellae”	*Dorsum sellae*	Dorso selar 58,5% (24/41)	Dorso da sela 41,5% (17/41)	Dorsum sellae 0,0%
“Anterior clinoid process”	*Processus clinoideus anterior (plur.: processus clinoidei anteriores)*	Processo clinóide anterior 92,7% (38/41)	Clinóide anterior 7,3% (3/41)	
“Posterior clinoid process”	*Processus clinoideus posterior (plur.: processus clinoidei posteriores)*	Processo clinóide posterior 92,7% (38/41)	Clinóide posterior 7,3% (3/41)	
“Posterior cranial fossa”	*Fossa cranii posterior*	Fossa craniana posterior 95,1% (39/41)		
“Clivus”	*Clivus*	Clivus 95,1% (39/41)	Clivo 2,45% (1/41)	Clivo 2,45% (1/41)

n.e., not exist.

a Chosen term.

b Adapted after discussion with the authors for better anatomical description.

### Phase 2. Iterative assessment

The most often suggested terms were reviewed by the authors under the supervision of authors with experience in anatomy (G.J. and H.Z.L.). A final list was created with up to three most relevant terms for each term.

### Phase 3. Final voting

An electronic survey was sent to each rhinologist through an online platform. The survey included multiple-choice options for each of the 126 terms, divided into the same eight previously defined blocks. All the rhinologists could choose only one term option in Portuguese for each term in English. The most voted term in Portuguese was chosen for each term in English. In cases of tie votes or when there was divergence of names for similar structures, these were discussed and decided by consensus.

### Phase 4. Writing of this article

A summarized list of terms in Portuguese was created together with this article for final approval by the authors.

## Results

All of the rhinologists accepted the invitation to participate. The final list of terms that were suggested and the voting frequency of the three terms voted on later are shown in Table 1. The proposed terms are underlined.

## Discussion

This study proposes a unified sinonasal anatomical terminology through the process of cross-cultural adaptation of the anatomical terms defined for the English language related to the nose and the paranasal cavities. The presence of researchers with experience in anatomical terms (G.J. and H.Z.L.) was important for the project adequacy.

The history of controversy regarding sinonasal anatomical terminology has existed for many years and can be exemplified by the use of the term infundibulum and semilunar hiatus to designate several lateral wall structures to the point that the abandonment of this terminology has been suggested in the past. In this terminology, we propose the use of terms that help to differentiate the anatomical structures so that eponyms are not utilized.^5^ We believe this favors the learning and the correct naming of the structures.

The vast majority of the results, as chosen by the rhinologists invited to participate in the study, were maintained. However, the results for some of the terms had to be better discussed to result in a uniform terminology for structures with similar names and a nomenclature adequacy with focus on surgical practice.

The nasal septum is a structure commonly divided into two parts, called bony and membranous parts. The uniformization of the term proposed by most authors for the membranous portion (of the nasal septum) was “Membranous part of the nasal septum”, by 53.7% [22/41]. On the other hand, despite the most often suggested term for “bony septum” [Anatomic terminology (AT): “pars osses septi nasi”] was “Bony Nasal Septum”, by 48.8% [20/41], we suggested the term “Bony part of the nasal septum” (34.1% [14/41]) to maintain uniformity in relation to the “membranous part of the nasal septum”.

We also suggested that for the term “inferior turbinate” (AT: *concha nasalis inferior*), the term “*concha inferior*” should be chosen (*concha inferior* 39% [16/41] vs. *concha nasal inferior*, 61% [25/41]). This term is most frequently used and will maintain the standardization in relation to the related structures: “Middle turbinate” [51.2% (21/41)], “Basal Lamella of Middle Turbinate” [53.7% (22/41)] “Superior turbinate” [48.8% (20/41)] and “Supreme nasal turbinate” [48.8% (20/41)].

“Nasal meatus” was also chosen instead of the term “meatus”, since there are other anatomical structures called “meatus” in other parts of the human body. Although this option received fewer votes, it would be the most appropriate one: “Inferior meatus” 53.7% (22/41) vs. “Inferior nasal meatus” 43.9% (18/41); “Middle meatus” 48.8% (20/41) vs. “Middle nasal meatus” 17.1% (7/41); “Superior meatus” 48.8% (20/41) vs. “Superior nasal meatus” 41.5% (17/41). Another recommended term that did not receive the most votes one was “supreme nasal meatus” instead of “nasal supreme meatus”. The most voted term, “nasal supreme meatus”, suggests that meatus is above the nose.

The term “olfactory fiber(s)”, 48.8% (20/41), although receiving the most votes, was also passed over for another term, because it is important to indicate that it is a “nerve” and to add the term “nerve”. We chose to use “olfactory nerve fibers”, 46.3% (19/41).

It was suggested that the site should be added to the term “accessory ostium”, 87.8% (36/41), since there are other accessory ostia in the body, and the term “accessory ostium of maxillary sinus” was suggested.

Despite the diverse voting, it was proposed that the term “ethmoidal cells” be replaced by “*complexo etmoidal*” following the English term “ethmoidal complex”. The “ethmoidal complex” would be subdivided into “anterior ethmoidal complex” and “posterior ethmoidal complex”; also diverging from the most voted terms: “anterior ethmoidal cells” and “posterior ethmoidal cells” for the same reason.

The term “frontal sinus drainage pathway” was also re-discussed consensually and we chose “*Via da drenagem do Seio frontal*” (26.8%; 11/41). Although it has been suggested that we use the term “frontal recess” (63.4%, 26/41) to designate this structure, the chosen term emphasizes that it is a different entity from the “frontal recess”, the proposed term of which is “*Recesso frontal*” (97.6%; 40/41). Although controversial, the terms “frontal recess” and “frontal sinus drainage pathway” are generally distinct entities. The frontal recess is generally defined as the most anterosuperior part of the ethmoid, inferior to the sinus opening.^1^ Its use as a synonym of “frontal sinus drainage pathway” is not appropriate, since the drainage pathway of the frontal sinus through the frontal recess is a complex one, altered by the configuration of the air cells within it and by the different connections of the uncinate process.^1^ It commonly includes the frontal recess, but is not constituted exclusively by it. Usually, the frontal recess is posteriorly delimited by the anterior wall of the ethmoidal bulla (if that is fixed at the base of the skull), antero-inferiorly by the agger nasi, laterally by the lamina papyracea and inferiorly by the terminal recess of the ethmoidal infundibulum, if present. The term “*ducto nasofrontal*” (from the anatomical terminology, “Ductus nasofrontalis”) was abandoned because the frontal sinus drainage pathway is not a true duct. The term “maxillary crest” (from the term “Lacrimal buldge” in English) was defined for this important structure as a point of reference for endoscopic dacryocystorhinostomy and is formed by the frontal process of the maxilla.

## Final consideration

We propose an adapted version in Portuguese of the “European Anatomical Terminology of the Internal Nose and Paranasal Sinuses”, that will help with the publication of technical announcements, scientific publications and the teaching of the internal anatomical terms of the nose and paranasal sinuses in Brazil.

## Conflicts of interest

The authors declare no conflicts of interest.
