# Erratum on “Anatomical terminology of the internal nose and paranasal sinuses: cross-cultural adaptation to Portuguese”^☆^

**DOI:** 10.1016/j.bjorl.2018.11.001

**Published:** 2019-01-01

**Authors:** Thiago Freire Pinto Bezerra, Aldo Stamm, Wilma Teresinha Anselmo-Lima, Marco Aurélio Fornazieri, Nelson D’Ávila Melo, Leonardo Balsalobre, Geraldo Pereira Jotz, Henrique Zaquia Leão, André Alencar Araripe Nunes, Alexandre Felippu, Antonio Carlos Cedin, Carlos D. Pinheiro-Neto, Diego Lima Oliveira, Eulalia Sakano, Eduardo Macoto Kosugi, Elizabeth Araújo, Fabiana Cardoso Pereira Valera, Fábio de Rezende Pinna, Fabrizio Ricci Romano, Francine Grecco de Melo Pádua, Henrique Faria Ramos, João Telles Jr., Leonardo Conrado Barbosa de Sá, Leopoldo Marques D’Assunção Filho, Luiz Ubirajara Sennes, Luis Carlos Gregório, Marcelo H. Sampaio, Marco César Jorge dos Santos, Marco Franca, Marcos Mocellin, Marcus Miranda Lessa, Melissa Ameloti G. Avelino, Miguel Tepedino, Nilvano Alves de Andrade, Otavio B. Piltcher, Renato Roithmann, Renata Mendonça Pilan, Roberto Campos Meireles, Roberto Eustáquio Guimarães, Rodrigo de Paula Santos, Rogério Pezato, Shirley Pignatari, Tatiana Telles Abdo, Victor Nakajima, Washington Almeida, Marcio Nakanishi, Richard L. Voegels

**Affiliations:** a Universidade Federal de Pernambuco (UFPE), Recife, PE, Brasil; b Universidade de São Paulo (USP), Faculdade de Medicina (FM), São Paulo, SP, Brasil; c Complexo Hospitalar Edmundo Vasconcelos, Centro de Otorrinolaringologia e Fonoaudiologia (COF), São Paulo, SP, Brasil; d Universidade de São Paulo (USP-RP), Faculdade de Medicina (FM), Ribeirão Preto, SP, Brasil; e Universidade Estadual de Londrina (UEL), Londrina, PR, Brasil; f Pontifícia Universidade Católica do Paraná (PUC-PR), Londrina, PR, Brasil; g Universidade Tiradentes (UNIT), Aracaju, SE, Brasil; h Universidade Federal do Rio Grande do Sul (UFRGS), Porto Alegre, RS, Brasil; i Universidade Federal do Ceará (UFC), Fortaleza, CE, Brasil; j Instituto Felippu de Otorrinolaringologia e Base do Crânio, São Paulo, SP, Brasil; k Beneficência Portuguesa de São Paulo, São Paulo, SP, Brasil; l Albany Medical Center, Albany, NY, EUA; m Hospital Memorial Arthur Ramos (HMAR), Maceió, AL, Brasil; n Universidade Estadual de Campinas (UNICAMP), Campinas, SP, Brasil; o Universidade Federal de São Paulo (UNIFESP), Escola Paulista de Medicina (EPM), São Paulo, SP, Brasil; p Hospital Israelita Albert Einstein, São Paulo, SP, Brasil; q Universidade Federal do Espírito Santo (UFES), Vitória, ES, Brasil; r Universidade do Estado do Rio de Janeiro (UERJ), Faculdade de Ciências Médicas, Rio de Janeiro, RJ, Brasil; s Instituto Paranaense de Otorrinolaringologia, Curitiba, PR, Brasil; t Faculdade de Enfermagem e Medicina Nova Esperança (FAMENE), João Pessoa, PB, Brasil; u Universidade Federal do Paraná (UFPR), Curitiba, PR, Brasil; v Universidade Federal da Bahia (UFBA), Faculdade de Medicina, Salvador, BA, Brasil; w Universidade Federal de Goiás (UFG), Goiânia, GO, Brasil; x Policlínica Botafogo, Rio de Janeiro, RJ, Brasil; y Fundação Bahiana para Desenvolvimento das Ciências, Salvador, BA, Brasil; z Universidade Luterana do Brasil, Faculdade de Medicina, Porto Alegre, RS, Brasil; aa Universidade Federal do Rio de Janeiro (UFRJ), Rio de Janeiro, RJ, Brasil; bb Universidade Federal de Minas Gerais (UFMG), Belo Horizonte, MG, Brasil; cc Universidade Estadual Paulista (UNESP), Botucatu, SP, Brasil; dd Hospital Otorrinos de Feira de Santana, Feira de Santana, BA, Brasil; ee Universidade de Brasília (UnB), Faculdade de Medicina, Programa de Pós-graduação em Ciências Medicas, Brasília, DF, Brasil

In the article “Anatomical terminology of the internal nose and paranasal sinuses: cross-cultural adaptation to Portuguese”, publicado no Braz J Otorhinolaryngol. 2018;84(6):677-686, on page 677, where it reads:

Thiago Freire Pinto Bezerra^a,b*^, Aldo Stamm^c^, Wilma Teresinha Anselmo-Lima^d^, Marco Aurélio Fornazieri^e,f^, Nelson D’Ávila Melo^g^, Leonardo Balsalobre^c^, Geraldo Pereira Jotz^h^, Henrique Zaquia Leão^h^, André Alencar Araripe Nunes^i^, Alexandre Felippu^j^, Antonio Carlos Cedin^k^, Carlos D. Pinheiro-Neto^l^, Diego Lima Oliveira^m^, Eulalia Sakano^n^, Eduardo Macoto Kosugi^o^, Elizabeth Araújo^h^, Fabiana Cardoso Pereira Valera^d^, Fábio de Rezende Pinna^b^, Fabrizio Ricci Romano^b^, Francine Grecco de Melo Pádua^p^, Henrique Faria Ramos^q^, João Telles Jr.^r^, Leonardo Conrado Barbosa de Sá^r^, Leopoldo Marques D’Assunção Filho^a^, Luiz Ubirajara Sennes^b^, Luis Carlos Gregório^o^, Marcelo H. Sampaio^n^, Marco César Jorge dos Santos^s^, Marco Franca^t^, Marcos Mocellin^u,s^, Marcus Miranda Lessa^v^, Melissa Ameloti G. Avelino^w^, Miguel Tepedino^r,x^, Nilvano Alves de Andrade^y^, Otavio B. Piltcher^h^, Renato Roithmann^z^, Renata Mendonça Pilan^b^, Roberto Campos Meireles^aa^, Roberto Eustáquio Guimarães^bb^, Rodrigo de Paula Santos^o^, Rogério Pezato^b,o^, Shirley Pignatari^o^, Tatiana Telles Abdo^b^, Victor Nakajima^cc^, Washington Almeida^dd^, Richard L. Voegels^b^

^*a*^ *Universidade Federal de Pernambuco (UFPE), Recife, PE, Brasil*

^b^ *Universidade de São Paulo (USP), Faculdade de Medicina (FM), São Paulo, SP, Brasil*

^c^ *Complexo Hospitalar Edmundo Vasconcelos, Centro de Otorrinolaringologia e Fonoaudiologia (COF), São Paulo, SP, Brasil*

^d^ *Universidade de São Paulo (USP-RP), Faculdade de Medicina (FM), Ribeirão Preto, SP, Brasil*

^e^ *Universidade Estadual de Londrina (UEL), Londrina, PR, Brasil*

^f^ *Pontifícia Universidade Católica do Paraná (PUC-PR), Londrina, PR, Brasil*

^g^ *Universidade Tiradentes (UNIT), Aracaju, SE, Brasil*

^h^ *Universidade Federal do Rio Grande do Sul (UFRGS), Porto Alegre, RS, Brasil*

^i^ *Universidade Federal do Ceará (UFC), Fortaleza, CE, Brasil*

^j^ *Instituto Felippu de Otorrinolaringologia e Base do Crânio, São Paulo, SP, Brasil*

^k^ *Beneficência Portuguesa de São Paulo, São Paulo, SP, Brasil*

^l^ *Albany Medical Center, Albany, NY, EUA*

^m^ *Hospital Memorial Arthur Ramos (HMAR), Maceió, AL, Brasil*

^n^ *Universidade Estadual de Campinas (UNICAMP), Campinas, SP, Brasil*

^o^ *Universidade Federal de São Paulo (UNIFESP), Escola Paulista de Medicina (EPM), São Paulo, SP, Brasil*

^p^ *Hospital Israelita Albert Einstein, São Paulo, SP, Brasil*

^q^ *Universidade Federal do Espírito Santo (UFES), Vitória, ES, Brasil*

^r^ *Universidade do Estado do Rio de Janeiro (UERJ), Faculdade de Ciências Médicas, Rio de Janeiro, RJ, Brasil*

^s^ *Instituto Paranaense de Otorrinolaringologia, Curitiba, PR, Brasil*

^t^ *Faculdade de Enfermagem e Medicina Nova Esperança (FAMENE), João Pessoa, PB, Brasil*

^u^ *Universidade Federal do Paraná (UFPR), Curitiba, PR, Brasil*

^v^ *Universidade Federal da Bahia (UFBA), Faculdade de Medicina, Salvador, BA, Brasil*

^w^ *Universidade Federal de Goiás (UFG), Goiânia, GO, Brasil*

^x^ *Policlínica Botafogo, Rio de Janeiro, RJ, Brasil*

^y^ *Fundação Bahiana para Desenvolvimento das Ciências, Salvador, BA, Brasil*

^z^ *Universidade Luterana do Brasil, Faculdade de Medicina, Porto Alegre, RS, Brasil*

^aa^ *Universidade Federal do Rio de Janeiro (UFRJ), Rio de Janeiro, RJ, Brasil*

^bb^ *Universidade Federal de Minas Gerais (UFMG), Belo Horizonte, MG, Brasil*

^cc^ *Universidade Estadual Paulista (UNESP), Botucatu, SP, Brasil*

^dd^ *Hospital Otorrinos de Feira de Santana, Feira de Santana, BA, Brasil*

It should read:

**Thiago Freire Pinto Bezerra**^**a**,**b**^∗**, Aldo Stamm**^**c**^**, Wilma Teresinha Anselmo-Lima**^**d**^**, Marco Aurélio Fornazieri**^**e**,**f**^**, Nelson D’Ávila Melo**^**g**^**, Leonardo Balsalobre**^**c**^**, Geraldo Pereira Jotz**^**h**^**, Henrique Zaquia Leão**^**h**^**, André Alencar Araripe Nunes**^**i**^**, Alexandre Felippu**^**j**^**, Antonio Carlos Cedin**^**k**^**, Carlos D. Pinheiro-Neto**^**l**^**, Diego Lima Oliveira**^**m**^**, Eulalia Sakano**^**n**^**, Eduardo Macoto Kosugi**^**o**^**, Elizabeth Araújo**^**h**^**, Fabiana Cardoso Pereira Valera**^**d**^**, Fábio de Rezende Pinna**^**b**^**, Fabrizio Ricci Romano**^**b**^**, Francine Grecco de Melo Pádua**^**p**^**, Henrique Faria Ramos**^**q**^**, João Telles Jr.**^**r**^**, Leonardo Conrado Barbosa de Sá**^**r**^**, Leopoldo Marques D’Assunção Filho**^**a**^**, Luiz Ubirajara Sennes**^**b**^**, Luis Carlos Gregório**^**o**^**, Marcelo H. Sampaio**^**n**^**, Marco César Jorge dos Santos**^**s**^**, Marco Franca**^**t**^**, Marcos Mocellin**^**u**,**s**^**, Marcus Miranda Lessa**^**v**^**, Melissa Ameloti G. Avelino**^**w**^**, Miguel Tepedino**^**r**,**x**^**, Nilvano Alves de Andrade**^**y**^**, Otavio B. Piltcher**^**h**^**, Renato Roithmann**^**z**^**, Renata Mendonça Pilan**^**b**^**, Roberto Campos Meireles**^**aa**^**, Roberto Eustáquio Guimarães**^**bb**^**, Rodrigo de Paula Santos**^**o**^**, Rogério Pezato**^**b**,**o**^**, Shirley Pignatari**^**o**^**, Tatiana Telles Abdo**^**b**^**, Victor Nakajima**^**cc**^**, Washington Almeida**^**dd**^**, Marcio Nakanishi**^**ee**^**, Richard L. Voegels**^**b**^

^a^ *Universidade Federal de Pernambuco (UFPE), Recife, PE, Brasil*

^b^ *Universidade de São Paulo (USP), Faculdade de Medicina (FM), São Paulo, SP, Brasil*

^c^ *Complexo Hospitalar Edmundo Vasconcelos, Centro de Otorrinolaringologia e Fonoaudiologia (COF), São Paulo, SP, Brasil*

^d^ *Universidade de São Paulo (USP-RP), Faculdade de Medicina (FM), Ribeirão Preto, SP, Brasil*

^e^ *Universidade Estadual de Londrina (UEL), Londrina, PR, Brasil*

^f^ *Pontifícia Universidade Católica do Paraná (PUC-PR), Londrina, PR, Brasil*

^g^ *Universidade Tiradentes (UNIT), Aracaju, SE, Brasil*

^h^ *Universidade Federal do Rio Grande do Sul (UFRGS), Porto Alegre, RS, Brasil*

^i^ *Universidade Federal do Ceará (UFC), Fortaleza, CE, Brasil*

^j^ *Instituto Felippu de Otorrinolaringologia e Base do Crânio, São Paulo, SP, Brasil*

^k^ *Beneficência Portuguesa de São Paulo, São Paulo, SP, Brasil*

^l^ *Albany Medical Center, Albany, NY, EUA*

^m^ *Hospital Memorial Arthur Ramos (HMAR), Maceió, AL, Brasil*

^n^ *Universidade Estadual de Campinas (UNICAMP), Campinas, SP, Brasil*

^o^ *Universidade Federal de São Paulo (UNIFESP), Escola Paulista de Medicina (EPM), São Paulo, SP, Brasil*

^p^ *Hospital Israelita Albert Einstein, São Paulo, SP, Brasil*

^q^ *Universidade Federal do Espírito Santo (UFES), Vitória, ES, Brasil*

^r^ *Universidade do Estado do Rio de Janeiro (UERJ), Faculdade de Ciências Médicas, Rio de Janeiro, RJ, Brasil*

^s^ *Instituto Paranaense de Otorrinolaringologia, Curitiba, PR, Brasil*

^t^ *Faculdade de Enfermagem e Medicina Nova Esperança (FAMENE), João Pessoa, PB, Brasil*

^u^ *Universidade Federal do Paraná (UFPR), Curitiba, PR, Brasil*

^v^ *Universidade Federal da Bahia (UFBA), Faculdade de Medicina, Salvador, BA, Brasil*

^w^ *Universidade Federal de Goiás (UFG), Goiânia, GO, Brasil*

^x^ *Policlínica Botafogo, Rio de Janeiro, RJ, Brasil*

^y^ *Fundação Bahiana para Desenvolvimento das Ciências, Salvador, BA, Brasil*

^z^ *Universidade Luterana do Brasil, Faculdade de Medicina, Porto Alegre, RS, Brasil*

^aa^ *Universidade Federal do Rio de Janeiro (UFRJ), Rio de Janeiro, RJ, Brasil*

^bb^ *Universidade Federal de Minas Gerais (UFMG), Belo Horizonte, MG, Brasil*

^cc^ *Universidade Estadual Paulista (UNESP), Botucatu, SP, Brasil*

^dd^ *Hospital Otorrinos de Feira de Santana, Feira de Santana, BA, Brasil*

^ee^ *Universidade de Brasília (UnB), Faculdade de Medicina, Programa de Pós-graduação em Ciências Medicas, Brasília, DF, Brasil*

